# Supplementary material for: Depressive Symptoms and Control of Emotions among Polish Women with Polycystic Ovary Syndrome
Source: Int J Environ Res Public Health. 2022 Dec 15;19(24):16871. doi: 10.3390/ijerph192416871 (PMC9779637; doi:10.3390/ijerph192416871)
Supplement: Supplementary file 1 [file ijerph-19-16871-s001.zip › ijerph-2052182-supplementary.pdf]

Depressive Symptoms and Control of Emotions among Polish Women with Polycystic Ovary Syndrome

Original questionnaire

*required

Demographics

1. How old are you?*

2. Where do you live?*

Mark only one answer.

a) Village

b) City up to 25.000 inhabitants

c) City in the range of 25-100.000 inhabitants

d) City in the range of 100-250.000 inhabitants

e) City over 250.000 inhabitants

3. What is your education?*

Mark only one answer.

a) Primary

b) Professional

c) Secondary

d) Higher

4. What is your body weight? (Please enter the result in kilograms)*

Open question.

5. What is your height? (Please enter the result in centimeters)*

Open question.

6. Have you tried to lose weight since your PCOS diagnosis?*

Mark only one answer.

a) Yes

b) No

7. If you answered YES to question 6, did you have difficulty losing weight?

Mark only one answer.

a) Yes

b) No

8. Do you undertake physical activity? (Physical activity for at least 30 min per day, which is not related to performing daily activities like work, shopping, and housework)*

Mark only one answer.

a) Yes

b) No

9. How often do you undertake physical activity?*

Mark only one answer.

a) 1-2 times a week

b) 3-4 times a week

c) Less than once a week

d) More than 4 times a week

e) I do not undertake physical activity

10. How long does your physical activity last on average? (Please enter the time in minutes) (If you are not active please enter 0)*

Open question.

11. What kind of activity do you do most often?*

Mark only one answer.

a) Running

b) Walks

c) Dance

d) Cycling

e) Fitness

f) Exercise in the gym

g) Swimming

h) I do not undertake physical activity

i) Other:

12. What other activities do you undertake? (You can select more than one response)

Mark all the correct answers.

a) Running

b) Walks

c) Dance

d) Cycling

e) Fitness

f) Gym exercises

g) Swimming

h) Other:

Menstrual cycle

13. What age did you have your first menstrual period?*

Mark only one answer.

a) 8 years

b) 9 years

c) 10 years

d) 11 years

e) 12 years

f) 13 years

g) 14 years

h) 15 years

i) 16 years

j) 17 years

k) 18 years

l) 19 years

m) 20 years

14. Are you currently pregnant? (IF YES, please provide answers to subsequent questions according to the state before pregnancy)*

Mark only one answer.

a) Yes

b) No

15. Is your cycle regular now? (The duration of the menstrual cycle is measured from the first day of bleeding to the first day of the next bleeding, ranging from 21-35 days, with an average of about 28 days)*

Mark only one answer.

a) Yes

b) No

16. What is the average duration of your cycle? Please enter the number of days.*

Open question.

17. How many days does your menstrual bleeding last? (Please specify with accuracy up to 1 day)*

Open question.

18. How intense is your menstrual bleeding?*

Mark only one answer.

a) Stingy

b) Moderate

c) Plentiful

19. Are your periods painful? (Whether during menstruation do you feel pain in your lower abdomen?) (0 means no pain, 10 means the worst pain you've ever felt?)*

Likert scale.

20. If your periods are painful, when do you feel pain?

Mark only one answer.

a) A few days before the onset of menstruation

b) For the first few days of menstruation

c) At the end of menstruation

d) For the entire duration of menstruation

21. In addition to painful contractions, do you have any other symptoms, such as:

Mark all the correct answers.

a) Nausea

b) Vomiting

c) Diarrhea

d) Headaches

e) Muscle aches

f) back pain

g) Breast pains

h) Hot flushes

i) Problems with sleeping

j) Mood changes

k) Fears, anxiety

l) Depression

m) Not applicable

22. Do you have bleeding in the middle of your cycle?*

Mark only one answer.

a) Yes

b) Sometimes, but not every cycle

c) No

23. How often do you see a gynecologist?*

Mark only one answer.

a) Once a year

b) Once every 2 years

c) Once every 3 years

d) Once every 4 years

e) I don't go

f) Other:

24. Do you use hormonal contraception? (pills, patches contraceptives, injections, intrauterine device, vaginal ring, contraceptive implant)*

Mark only one answer.

a) Yes

b) No

25. If in Q. 24 you marked the answer YES, then: Before using of hormonal contraception had regular cycles?

Mark only one answer.

a) Yes

b) Not

c) Not applicable

26. Have you ever been pregnant?*

Mark only one answer.

a) Yes

b) No

27. Do you have children?*

Mark only one answer.

a) Yes

b) No

28. If in Q. 27 you answered YES, how many children do you have?

Mark only one answer.

a) 1

b) 2

c) 3

d) 4

e) 5

f) 6

g) 7

h) 8

29. When trying to conceive, did you have regular intercourse (2-4 times a week) over a 12-month period without using contraception?*

Mark only one answer.

a) Yes

b) No

c) So far, I have not tried for offspring

30. What was the result of your maternity efforts?

Mark only one answer.

a) I got pregnant

b) I failed to get pregnant

31. Have you ever been treated for infertility?*

Mark only one answer.

a) Yes

b) No

PCOS

32. How many years ago were you diagnosed with PCOS (Polycystic Ovary Syndrome) by a doctor?*

Mark only one answer.

a) <1 year

b) 1-5 years

c) >5 years

33. Has anyone in your family suffered from PCOS (mother, sister, aunt, cousin)?*

Mark only one answer.

a) Yes

b) No

34. If you answered YES in question 33, who from your family member suffered from PCOS?

Mark all the correct answers.

a) Mom

b) Sister

c) Auntie

d) Cousin

e) Grandmother

f) Not applicable

35. Have you had any of the following symptoms? (can be ticked a few)*

Mark all the correct answers.

a) Acne

b) Seborrhea, oily hair, facial skin

c) Hirsutism, i.e. the presence of an excessive amount of mature hair in androgen-sensitive areas of the body, including: on the chest, around the nipples, above the upper lip, on the chin, on the stomach and back, on the inner surface of the thighs

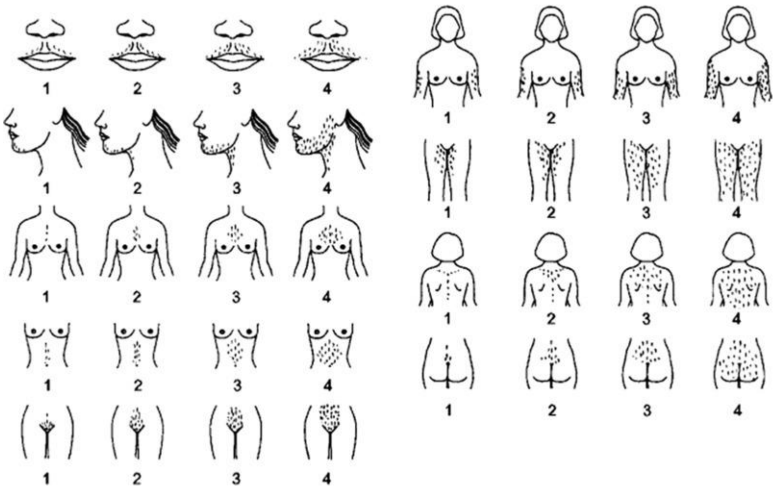

d) Male pattern baldness – loss hair on top of head or receding hairline

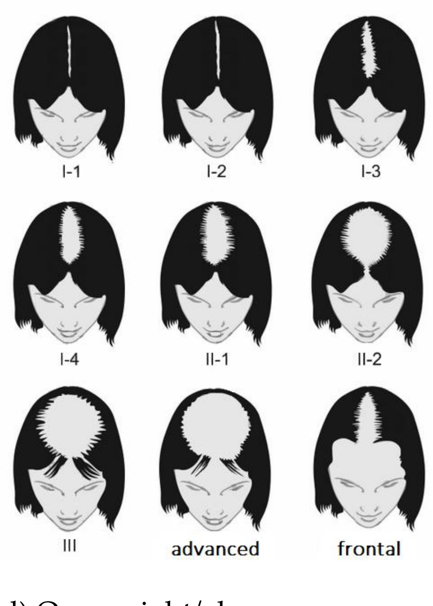

d) Overweight/obese

e) Infertility

f) Not applicable

36. Have you ever had in the past or currently elevated levels of androgens (androstenedione, testosterone)?*

Mark only one answer.

a) Yes

b) Not

c) I do not know/I do not remember

37. Were they present in the past or currently in the ultrasound examination polycystic ovaries?*

Mark only one answer.

a) Yes

b) No

c) I do know/I do not remember

38. Do you have any of the following ailments? (you can mark several)*

Mark all the correct answers.

a) Hypertension (blood pressure ≥140/≥90 mmHg)

b) Diabetes

c) Insulin resistance

d) Prediabetes - IGT (impaired glucose tolerance) and IFG (impaired fasting glucose)

f) Lipid disorders (increased total cholesterol, LDL or triglycerides)

g) Sleep apnea

h) Dark skin in the armpits, nape and groin area

i) Not applicable

39. After being diagnosed with PCOS, have you made any attempt to the following behaviors? (you can select several)*

Mark all the correct answers.

a) Weight reduction

b) Increased physical activity

c) Avoiding foods with a high glycemic index

d) Caloric restricted diet

e) Myo-inositol supplementation

f) Vitamin D supplementation

g) Not applicable

40. Are you taking medication for PCOS?*

Mark only one answer.

a) Yes

b) No

41. If you answered YES to the previous question, what medications do you take? (please also include drugs that improve mood, e.g. cital, trazodone etc.)

Open question.

The next questions included the standardized Beck Depression and CECS questionnaires.
